# Takotsubo Cardiomyopathy: A Possible Rare Complication of Guillain-Barré Syndrome

**DOI:** 10.7759/cureus.40083

**Published:** 2023-06-07

**Authors:** Khalid H Mohamed, Adetola F Oshikoya, Kapil Kumar, Chinyere L Anigbo, Polasu Sri Satya Sai Prashanth, Alaa S Mohamed, Muhammad Haseeb, Hira Nasir

**Affiliations:** 1 Neurology, Sheffield Teaching Hospitals NHS Foundation Trust, Sheffield, GBR; 2 Internal Medicine, Near East University, Nicosia, CYP; 3 Internal Medicine, General Hospital Odan Lagos, Lagos, NGA; 4 Medicine and Surgery, Liaquat National Hospital and Medical College, Karachi, PAK; 5 Internal Medicine, University of Nigeria, Enugu, NGA; 6 Internal Medicine, M S Ramaiah Medical College, Bangalore, IND; 7 Neurology, Augusta University, Augusta, USA; 8 Internal Medicine, Allama Iqbal Medical College, Lahore, PAK; 9 Internal Medicine, Mount Sinai Hospital, Brooklyn, USA; 10 Internal Medicine, Mayo Hospital, Lahore, PAK

**Keywords:** takotsubo cardiomyopathy, gbs complication, gbs, guillain barre syndrome, reversible cardiomyopathy

## Abstract

Dysautonomia is a common and severe complication of Guillain-Barré syndrome (GBS), which may manifest as cardiac arrhythmias, labile blood pressure, diaphoresis, and changes in gastrointestinal motility. Takotsubo cardiomyopathy (TCM) is a life-threatening manifestation of dysautonomia in patients with GBS, which is not widely underlined in the literature. The association between GBS and TCM has been well-documented in previous studies; however, there are few reported cases with GBS who developed TCM following their diagnosis with GBS. In this case report, we will discuss our experience treating a 59-year-old female patient who became hemodynamically unstable while recovering from an acute GBS infection. She was diagnosed with TCM after undergoing an echocardiogram and coronary angiogram ruling out thrombotic or obstructive coronary disease and myocarditis.

## Introduction

Guillain-Barré syndrome (GBS) is an immune-mediated, acute neuromuscular disorder characterized by inflammatory demyelinating polyneuropathy, mainly affecting the peripheral nervous system (PNS) and presenting as sudden-onset, rapidly progressive symmetric ascending paresis and sensory paresthesia usually preceded by an infectious trigger [1]. Dysautonomia is a common life-threatening adverse event of GBS, presenting in almost 70% of the patients [2]. Dysautonomia is typically transient and may manifest as cardiac arrhythmias, labile blood pressure, diaphoresis, and changes in gastrointestinal motility [3]. Takotsubo cardiomyopathy (TCM) is a life-threatening manifestation of dysautonomia in patients with GBS, which is not widely underlined in the literature. The association between GBS and TCM has been well-documented in previous studies; however, there are few reported cases of patients with GBS who developed TCM following their diagnosis of GBS [3-7]. In this case report, we will discuss our experience treating a 59-year-old female patient who became hemodynamically unstable while recovering from an acute GBS infection.

## Case presentation

A 59-year-old female was brought to the emergency department with progressive bilateral lower limb weakness, numbness, and upper limb paresthesia, followed by dysarthria. Weakness was gradual in onset, progressive, and started from the lower limbs with no aggravating and relieving factors. The weakness was ascending, followed by upper limb involvement for the last three days and dysarthria, which developed the previous night. She had no significant medical or family history of any disease, psychosocial, or trauma history. She had no drug allergies and was not taking any medication during the presentation. On further evaluation, she had an upper respiratory tract infection two weeks ago.

On examination, she was afebrile, hemodynamically stable, and oriented to time, place, and person. On neurological examination, her power was 1/5 in the lower limbs and 4/5 in the upper limb, with absent tendon reflexes and paresthesia. The cranial nerve examination was normal, and she had no signs of meningeal irritation. Respiratory and cardiovascular examinations were unremarkable. Brain magnetic resonance imaging (MRI) was normal except for age-related changes. Cerebrospinal fluid analysis revealed albuminocytologic dissociation with an elevated protein level of 89 mg/dl (<60 mg/dl) and normal cell count. Electrodiagnostic studies revealed remarkable slow conduction velocities and temporal dissociation consistent with severe sensorimotor polyneuropathy with demyelinating features suggestive of GBS. Her initial laboratory evaluations were within normal range. She was commenced on intravenous immunoglobulin (400 mg/kg) for five days and plasmapheresis on alternate days.

**Figure 1:**
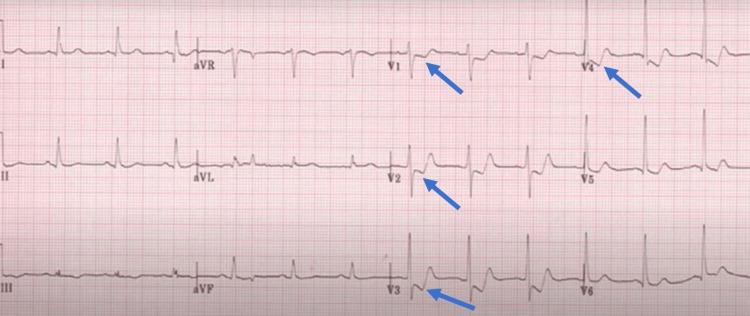
Electrocardiogram revealing T wave inversion in leads V1-V4

**Figure 2:**
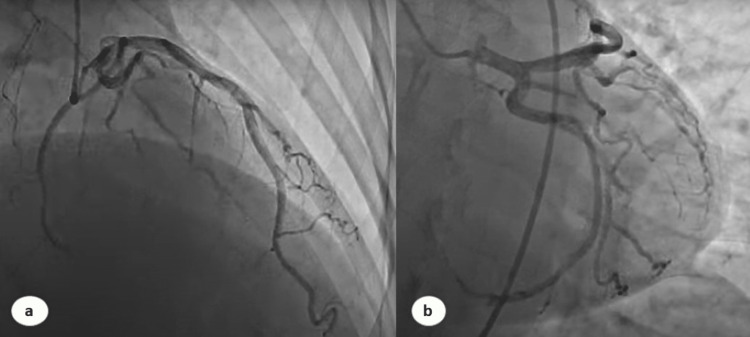
Coronary artery angiogram demonstrating normal right (a) and left (b) coronary arteries and their associated branches

The following day, she developed progressive respiratory failure because of a mucus plug and poor respiratory effort, which required intensive care unit (ICU) admission and elective intubation. Despite fluid load, she became hemodynamically unstable with persistent tachycardia and labile blood pressure. She underwent chest computed tomography to rule out pulmonary embolism, which was normal. Electrocardiogram (EKG) revealed sinus tachycardia with T wave inversion in anterolateral and septal leads (Figure 1). An urgent echocardiogram revealed mild to moderate anterolateral hypokinesia with an estimated ejection fraction of 30%. Laboratory evaluations, including blood culture and viral serology, were unremarkable except for an elevated troponin I of 6.4 ng/ml (0-0.04). Urgent coronary angiography was normal, with no significant obstructive or thrombotic arterial stenosis, suggesting stress cardiomyopathy with a Takotsubo risk score of 59 (Figure 2). She was managed with broad-spectrum antibiotics, hydrocortisone, norepinephrine (80 ug/minute), dobutamine (15 ug/kg/minute), and additional fluids. Her blood pressure started improving gradually, and she returned to diuresis. Additional furosemide was added due to positive fluid balance. Over the next 48 hours, her norepinephrine and dobutamine were tapered, and she was hemodynamically stable gradually. Metoprolol was added to her regimen due to persistent tachycardia. Her repeat echocardiogram on Day 11 was normal, with an improved ejection fraction.

**Figure 3:**
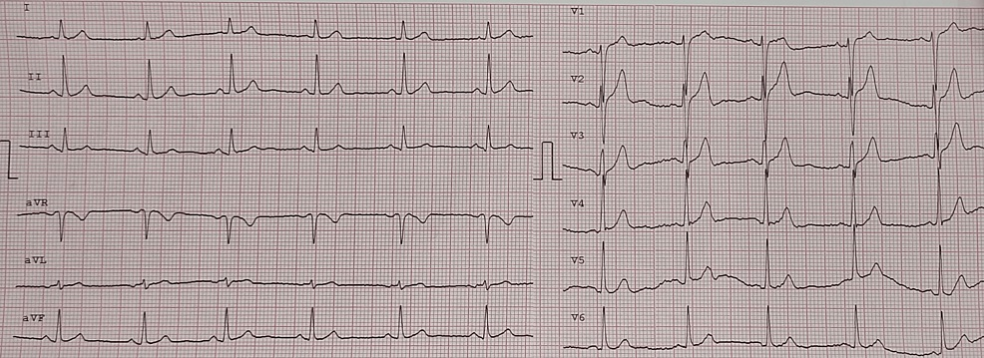
Electrocardiogram demonstrating normal electric activity of the heart

She failed several continuous positive airway pressure (CPAP) trials and underwent a tracheostomy on Day 14 because of difficult weaning. She was discharged on Day 28 with a referral to a long-term care facility for further physical rehabilitation. She still had motor neuropathy, and her ECG and echocardiogram were unremarkable (Figure 3).

## Discussion

TCM is also called stress cardiomyopathy, or broken heart syndrome, named after a Japanese jar used to capture octopuses, and has a round bottom with a tight neck, resembling an echocardiographic picture of a patient in an echocardiogram, which is called apical ballooning [8]. As further was been published, it became evident that wall motion abnormalities in TCM have been no longer confined to the apex but could additionally affect more than one segment of the left ventricle. Mayo Clinic criteria to diagnose TCM include mid-segment transitory hypokinesia, dyskinesia, or akinesia of the left ventricle along with or without the involvement of the apex of the heart; localizable wall motility disorders that are not linked to one specific cardiac vessel supply region; the presence of stressful stimulus; no evidence of thrombotic or obstructive coronary artery disease; new EKG changes like ST-segment elevation or T wave abnormalities, and absence of myocardial inflammation or pheochromocytoma [9].

The pathophysiology of TCM in GBS is not well-understood, and it is believed to be related to a complex interplay of factors, including autonomic dysfunction, catecholamine release, and inflammation [4-6]. GBS is thought to be caused by an autoimmune response that results in damage to the peripheral nerves. This damage can lead to autonomic dysfunction, which can affect the function of the heart. The autonomic nervous system regulates the heart rate, blood pressure, and other cardiovascular functions. When the autonomic nervous system is affected by GBS, it can result in abnormalities in these functions, including an increased release of catecholamines such as adrenaline and noradrenaline [10]. The release of catecholamines can cause direct damage to the heart muscle cells, leading to a temporary weakening of the left ventricle. This weakening of the left ventricle can result in the characteristic ballooning of the ventricle seen in TCM. In addition, the release of catecholamines can lead to vasoconstriction, reducing blood flow to the heart and exacerbating cardiac dysfunction. Inflammation is also believed to play a role in developing TCM in GBS [11]. Inflammatory cytokines can cause direct damage to the heart muscle cells and impair their function. Additionally, inflammation can lead to oxidative stress, further damaging the heart muscle cells and exacerbating cardiac dysfunction [12].

Individualized treatment is crucial for each patient, and using norepinephrine may be counterproductive [5]. Beta-blockade is recommended in cases of dynamic mid-ventricular obstruction, which can be detected through echocardiography [11]. Angiotensin-converting enzyme inhibitor (ACE) inhibition may also reduce afterload in hemodynamically stable patients [13]. In cases of suspected TCM in GBS, echocardiography should be performed to rule out acute coronary disease and myocarditis [14,15]. In our case study, the lack of cardiovascular risk factors, unremarkable creatine kinase levels, and ECG and echocardiogram abnormalities that could not be explained by regional coronary hypoperfusion/ischemia indicated that coronary artery disease was unlikely.

Due to autonomic dysfunctions like acute coronary syndromes, labile blood pressure, myocarditis, or tachyarrhythmias, TCM is hard to discern from more common cardiovascular adverse events of GBS [16]. Our patient was diagnosed with GBS and developed TCM. A massive release of catecholamines after anesthesia induction most likely contributed to TCM because of rapid hemodynamic instability and respiratory deterioration. Additionally, aggravated catecholamines accumulation after norepinephrine infusion may have caused her further myocardial dysfunction.

## Conclusions

Although rare, TCM is a life-threatening complication of GBS that requires urgent evaluation and management due to high morbidity and mortality. Sudden onset hemodynamic instability and new-onset EKG raise the suspicion of TCM in patients with GBS and must be ruled out using serial EKGs, echocardiography, and coronary angiography. Early diagnosis and management improve the prognosis of the patient.
